# Irreconcilable Conflict: The Tobacco Industry and the Public Health Challenge of Tobacco Use

**DOI:** 10.1371/journal.pmed.1001457

**Published:** 2013-05-28

**Authors:** Thomas E. Novotny

**Affiliations:** Graduate School of Public Health, San Diego State University, San Diego, California, United States of America

## Abstract

Thomas Novotny reflects on new research by Stanton Glantz and colleagues and the implications of their findings for engaging the tobacco industry on tobacco product regulation.

**Linked Research Article**

This Perspective discusses the following new study published in *PLOS Medicine*:

Tan CE, Kyriss T, Glantz SA (2013) Tobacco Company Efforts to Influence the Food and Drug Administration-Commissioned Institute of Medicine Report *Clearing the Smoke*: An Analysis of Documents Released through Litigation. PLoS Med 10(5): e1001450. doi:10.1371/journal.pmed.1001450

Stanton Glantz and colleagues investigate efforts by tobacco companies to influence *Clearing the Smoke*, a 2001 Institute of Medicine report on harm reduction tobacco products.

In this week's *PLOS Medicine*, Stanton Glantz and colleagues have conducted detailed historical research on the involvement of the tobacco industry, or at least its influence, in the preparation of an important Institute of Medicine (IOM) report (*Clearing the Smoke*) commissioned by the US Food and Drug Administration (FDA) in 2001 [1]. This report served as a backdrop for consideration of FDA regulatory authority over tobacco products at that time, and FDA authority has subsequently been enacted through the Family Smoking Prevention and Tobacco Control Act of 2009 (FSPTCA) [2]. We have seen several important developments in the United States regarding the tobacco industry since the 2001 IOM process, which seemed to then recognize that industry as a “stakeholder” and not as the ruthless and well-funded opponent of tobacco control it has been for decades. The authors here have rightfully pointed out that this industry has been cited in two federal courts for violating the Racketeer Influenced and Corrupt Organizations (RICO) Act during 50 years' of public deception about the harmfulness (and in addition the alleged *reduced harm*) of cigarettes and new tobacco products [3]. This fact cannot be ignored in any current or future regulatory approach to tobacco products. To paraphrase former Surgeon General C. Everett Koop, cigarettes were and still are the only consumer product that can kill you when used as directed.

In support of their IOM interventions in 1999–2001, tobacco companies recruited and employed paid “experts” to provide input to the deliberations and contributed well-crafted criteria for the development of “reduced harm” products to the IOM committee members. Presentations to the IOM were carefully vetted by industry lawyers in order to protect the business of selling tobacco and to fend off meddlesome public health regulations over product development. Glantz and colleagues show in great detail how clever, coordinated, and manipulative the big tobacco companies and their lawyers were in their efforts to insert their agenda into the IOM proceedings. It was clear to the industry that tobacco regulation was going to happen someday, and thus the tobacco companies needed to appear somehow to support the regulatory development process as good corporate citizens, despite their continued dedication to profiting from selling lethal products.

The public health approach to regulatory intervention is normally very inclusive, bringing all stakeholders to the table to present their perspectives, to argue about the impacts of the interventions on their organizations, and to find compromises that work for the greater good of all those involved. However, the tobacco industry is very good at what it does in terms of obfuscating the truth about the harm of tobacco use, dividing the public health community over harm reduction approaches, and befuddling critically important regulatory processes, even those that the FDA is now trying to implement under the FSPTCA. History has shown that the tobacco industry is NOT a stakeholder in public health and thus must not be treated as such.

In the United States today, the FDA has been endowed with an enormously challenging and historical opportunity to finally establish effective regulations over the tobacco industry in its pursuit of new users and its deception of current users. The FSPTCA allows the FDA to require standards for tobacco products to protect public health, bans cigarettes with flavors (except for menthol), restricts sales of tobacco products especially to youth, prohibits advertising and promotion of tobacco products, and establishes procedures regarding the marketing of new “modified risk” products. Tobacco products, including the addictive nicotine in them, remain as legally sold “adult” consumer goods, and the tobacco industry is committed to maintaining the consumer acceptability of these goods. The FDA now has the authority to make those goods LESS attractive to new users (i.e., children) and to fully inform smokers about the actual harms of these products through graphic labeling and prohibiting the use of “light” and “low tar” wording on packages. It has not been easy for the FDA to exert this authority, whether obstructed by incessant legal interventions (such as with the originally proposed graphic warning labels) or restrained by lines of authority in Congress or the Administration.

The FDA's deliberative process on tobacco product regulation, including that of its Tobacco Products Scientific Advisory Committee, need not incorporate the input of the tobacco industry, its experts, or in particular, its lawyers. The industry can certainly provide its commentary in this government regulatory process, and it has all the resources it needs to do so. However, it should never be treated as a stakeholder because it is unlikely that the industry will ever be part of the solution to the public health challenge of tobacco use. The profits from selling cigarettes and alternative tobacco products are simply too great for the tobacco industry to simply fade into history. Thus, the public health community needs to do what it does best: to rally popular support for strong, science-based approaches to prevention of tobacco use, to expose the truths about the harms of tobacco use to current users, and to support government agencies in carrying out their legislatively mandated duties to protect public health. This important moment in history must not be neglected, nor should the irreconcilable conflict between the public health community and the global tobacco industry be misunderstood.

## Abbreviations

FDA: US Food and Drug Administration
FSPTCA: Family Smoking Prevention and Tobacco Control Act of 2009
IOM: Institute of Medicine
